# Teaching gene-environment interaction concepts with narrative vignettes: Effects on knowledge, stigma, and behavior motivation

**DOI:** 10.1371/journal.pone.0300452

**Published:** 2024-05-09

**Authors:** Junhan Chen, Alison Jane Martingano, Siri Ravuri, Kaylee Foor, Christopher Fortney, Susan Carnell, Sapna Batheja, Susan Persky

**Affiliations:** 1 Social and Behavioral Research Branch, National Human Genome Research Institute, Bethesda, MD, United States of America; 2 University of Wisconsin-Green Bay, Sheboygan, WI, United States of America; 3 School of Medicine, Johns Hopkins University, Baltimore, MD, United States of America; 4 College of Public Health, George Mason University, Fairfax, VA, United States of America; International Medical University, MALAYSIA

## Abstract

Gene-environment interaction (GxE) concepts underlie a proper understanding of complex disease risk and risk-reducing behavior. Communicating GxE concepts is a challenge. This study designed an educational intervention that communicated GxE concepts in the context of eating behavior and its impact on weight, and tested its efficacy in changing knowledge, stigma, and behavior motivation. The study also explored whether different framings of GxE education and matching frames with individual eating tendencies would result in stronger intervention impact. The experiment included four GxE education conditions and a control condition unrelated to GxE concepts. In the education conditions, participants watched a video introducing GxE concepts then one of four narrative vignettes depicting how a character’s experience with eating hyperpalatable or bitter tasting food (reward-based eating drive vs. bitter taste perception scenario) is influenced by genetic or environmental variations (genetic vs. environmental framings). The education intervention increased GxE knowledge, genetic causal attributions, and empathetic concern. Mediation analyses suggest that causal attributions, particularly to genetics and willpower, are key factors that drive downstream stigma and eating behavior outcomes and could be targeted in future interventions. Tailoring GxE education frames to individual traits may lead to more meaningful outcomes. For example, genetic (vs. environmental) framed GxE education may reduce stigma toward individuals with certain eating tendencies among individuals without such tendencies. GxE education interventions would be most likely to achieve desired outcomes such as reducing stigma if they target certain causal beliefs and are strategically tailored to individual attributes.

## Introduction

Complex diseases, which present a significant public health challenge in the United States, often result from interactions between one’s genome and their environment [1]. With the growing influence of genomics technologies on disease risk prediction and prevention [2], and their potential to encourage risk-reducing behaviors [3], effectively conveying gene-environment interaction (GxE) concepts is increasingly important. At the same time, GxE remains a difficult concept to communicate, and new approaches are needed to increase GxE knowledge and support knowledge application to relevant health and social domains.

GxE refers to the notion that the phenotypic expression of traits, diseases, and other features depends on multiple genes, environmental factors, and their interactions [4]. GxE concepts underlie a proper understanding of complex disease risk and risk-reducing behavior, which is crucial for individual health decision-making and is especially helpful for those at high genetic risk [5–7]. Better understanding of GxE has been associated with less deterministic thinking about health outcomes, heightened consideration of environmental and behavioral factors that influence health conditions, stronger motivation to adopt risk-reducing behaviors, and reduced stigmatization of individuals with certain health conditions [8–10]. Given their importance, GxE concepts have long been targeted for education across public health, healthcare, and science education domains [6, 7, 11].

Successfully communicating GxE concepts has proven challenging, with school curricula [6], web-based materials [12], and media portrayals [13] often falling short of stated goals. Previous attempts have often either emphasized the influence of one factor while diminishing others or have presented genetic and environmental factors as independent causes of health conditions. This is consistent with the notion that individuals tend to consider genetic and environmental factors separately, switching between genetics- and environment-dominant models depending on context [14]. This makes communicating the two causal factors as a joint model particularly challenging. Exploratory educational intervention studies suggest that using metaphors may improve GxE knowledge, but the effectiveness of this approach may depend on factors such as the delivery approach and the complexity of the metaphors [15, 16]. Thus, novel educational interventions are needed to effectively teach GxE concepts.

### Communicating GxE influences on eating behavior

This study focuses on communicating GxE concepts in the context of eating behavior and its influence on weight. Eating behavior is influenced by interactions between genes and environment [17–20]. Eating behaviors are familiar and concrete to most individuals. Therefore, communicating GxE concepts in the context of eating behavior may be more easily understood by general audiences and may improve GxE knowledge and modify causal attributions for eating behaviors. However, eating behavior traits have not been explored as a basis for teaching GxE concepts.

In addition, the potential downstream effects of communicating GxE concepts related to eating behavior remain unexplored. Here the downstream outcomes include empathy, weight bias, and behavior change outcomes that may stem from changes to causal beliefs. Studies examining the influence of genetic attributions on eating behavior and those investigating GxE causal attributions for weight, suggest potential influences on both motivation for health-promoting behaviors and on stigmatization and empathy related to weight status. Overall, the literature suggests that causal beliefs are expected to mediate the influence of communicating GxE concepts on downstream outcomes as they have previously been associated with stigma [21, 22] and behavior change motivations [23]. GxE explanations can sometimes reduce blame and negative attitudes arising from purely environmental or behavioral explanations for weight [24–26]. A recent study that directly tested the mediation mechanism found that a GxE education intervention related to eating behaviors reduced weight stigma and increased empathy through increasing genetic causal beliefs [10].

However, GxE education aiming to alter causal beliefs may not always lead to the intended outcomes. Previous work has uncovered undesirable negative effects of GxE education, such as disengagement from risk information [26]. Genetic attributions for eating behaviors have sometimes led to fatalistic beliefs and maladaptive dietary behaviors [27, 28]. This highlights the need for GxE educational interventions that are easy to understand, applicable to everyday life, and that avoid potential misinterpretation. Moreover, given the key role of moderators in determining the influence of causal attributions, it is essential to consider the situations under which GxE education can be most successful in supporting learning, prompting empathy, reducing stigma, and in encouraging rather than discouraging health-promoting behavior.

### Framing in GxE education

When teaching GxE concepts, examples are typically framed either in terms of genetics (i.e., how a single environment might differentially affect individuals with two different genotypes) or environment (i.e., how a single genotype might manifest in two different environments). Framing can influence message interpretation and message effects [5, 29], and certain frames might better match specific educational outcomes [25, 30]. We propose that environmental framing may better encourage behavior change motivation, while genetic framing may better increase empathy and reduce stigma.

Environmental framing highlights how variations or purposeful changes in the environment can result in different health outcomes for an individual with a given genotype. The Health Belief Model [31] suggests that communicating about modifiable environmental factors provides a cue to action, which may prompt individuals to consider making such modifications with increased self-efficacy [32]. Consistent with this theoretical prediction, emphasizing the environment could increase perceived controllability of one’s eating tendencies and self-efficacy, and thereby increase intention for health behavior change [22, 25, 33]. Indeed, deterministic outcomes noted in previous literature are often interpreted as responses to the genetic component of the GxE message [27].

On the other hand, framing which emphasizes unmodifiable genetic influences on eating behavior in GxE education might be more effective for eliciting empathy and reducing stigmatizing attitudes. As suggested by Weiner’s attribution theory [34], higher genetic attributions are associated with less perceived personal responsibility for one’s condition [35, 36], which can increase sympathy and prosocial behaviors toward stigmatized individuals. Emphasizing genetic factors related to weight is often associated with less expression of weight-based stigma and bias [22, 24, 37, 38], and more willingness to help people with obesity [25].

### Tailoring in GxE education

Tailoring and targeting interventions to key sub-populations has long been considered best practice in health communication [39]. Tailored communication is designed to be relevant to individuals’ experiences, attitudes, and concerns, thereby increasing message effectiveness [39]. Studies suggest that the effectiveness of obesity education interventions, for example, is dependent on individuals’ education level [24], pre-existing causal beliefs [22], emotional state [40], and personal history of the health condition [41]. In the context of GxE education, message tailoring based on individual traits and attributes has been understudied, despite evidence supporting tailoring effects in other contexts.

The current study provides education in the context of two specific eating behavior tendencies—reward-based eating drive (RBED) and bitter taste perception (BTP). RBED is an “excessive drive to eat that results from feelings of lack of control, diminished satiety, and preoccupation with food” [42, p. 2]. With high RBED trait, individuals are more likely to consume less nutritious foods, particularly those rich in sugar, fat, and salt, such as highly-processed foods [43, 44]. BTP describes one’s sensitivity to the bitter taste of certain compounds in food [45]. Individuals who are more sensitive to bitter taste tend to dislike bitter foods such as certain types of vegetables [45]. Both tendencies predispose individuals toward less healthy eating behavior and are associated with reduced dietary self-efficacy [43–46].

Whether or not an individual has a predisposition toward RBED and/or BTP is an important factor to consider for tailoring an educational intervention. Illustrating GxE concepts in a scenario related to an eating behavior trait one experiences should be more relevant and directly address any existing negative beliefs or habits [39]. In addition, although both traits relate to genetic predispositions, RBED is culturally associated with a lack of willpower or behavioral control [42], while the genetic link to bitter taste receptors is more straightforward [47]. RBED may also be viewed more negatively than BTP [48] due to assumptions that it is more controllable.

Therefore, individuals with high RBED trait may relate more to environmental framing, while genetic framing may be better understood by individuals with high BTP trait. Previous work has also shown that for some eating behavior traits, including BTP, whether or not an individual exhibits that trait determines how genetic causal attributions for the trait influence dietary confidence [46]. These examples demonstrate the potential for one’s own eating behavior traits to influence responses to GxE education. Understanding tailoring effects on key outcomes can inform practical approaches in future intervention design.

### The present study

The present study tested the effectiveness of communicating GxE concepts through a combination of an educational video and experiential narrative vignettes. In these vignettes, individuals were asked to imagine themselves in food-relevant scenarios with genetic and environmental risk factors for less healthy eating behaviors, followed by an alternative scenario where either the genetic or the environmental factor was changed. The vignettes were described from an experiential perspective (e.g., “imagine your genes make you more likely to notice and pay attention to delicious foods in your environment”.) This approach was used because first-person narratives can make scientific concepts more relevant and accessible, enhancing content understanding and retention of the educational content [49, 50]. Moreover, perspective-taking can be helpful in generating empathy and reducing stigma [10, 51, 52].

The study design experimentally varied framing (genetic vs. environmental) and the character’s experience with eating hyperpalatable or bitter food (RBED vs. BTP) and included a control condition unrelated to GxE concepts. We preregistered hypotheses with Open Science Foundation (https://osf.io/zmbqu?view_only=d22201aa09294414b11fbb1ce9a3969b) and added exploratory research questions. Causal beliefs, perspective taking, and confidence were not included in the preregistration. We added these outcomes because they are conceptually related to the outcomes proposed in hypotheses (i.e., GxE knowledge, empathetic concern, and self-efficacy).

H1: GxE educational vignettes will result in
  - better GxE knowledge (i.e., knowledge recall, knowledge application) and stronger genetic and environmental causal beliefs,
  - greater empathy toward vignette characters (i.e., empathetic concern and perspective taking),
  - and less weight bias (i.e., weight stigma and weight stereotyping) than the control condition.
H2: GxE educational vignettes with an environmental framing will result in greater behavior change motivation (i.e., confidence, self-efficacy, and intention to change diet) compared to the control condition (H2a) and to the genetic framing condition (H2b).
H3: GxE educational vignettes with a genetic framing will result in greater empathy toward vignette characters (i.e., empathetic concern and perspective taking) and less weight bias (i.e., weight stigma and weight stereotyping) compared to the control condition (H3a) and to vignettes with an environmental framing (H3b).
RQ1: Does the relative effectiveness of framing and scenario type vary for individuals who are high vs low in the relevant eating behavior traits (i.e., RBED and BTP)?
RQ2: Are effects of the intervention on empathy, stigma, and health behavior outcomes mediated by causal beliefs, and do mediation models reveal any indirect effects in the absence of direct effects?

## Methods

### Design and procedure

**Fig 1:**
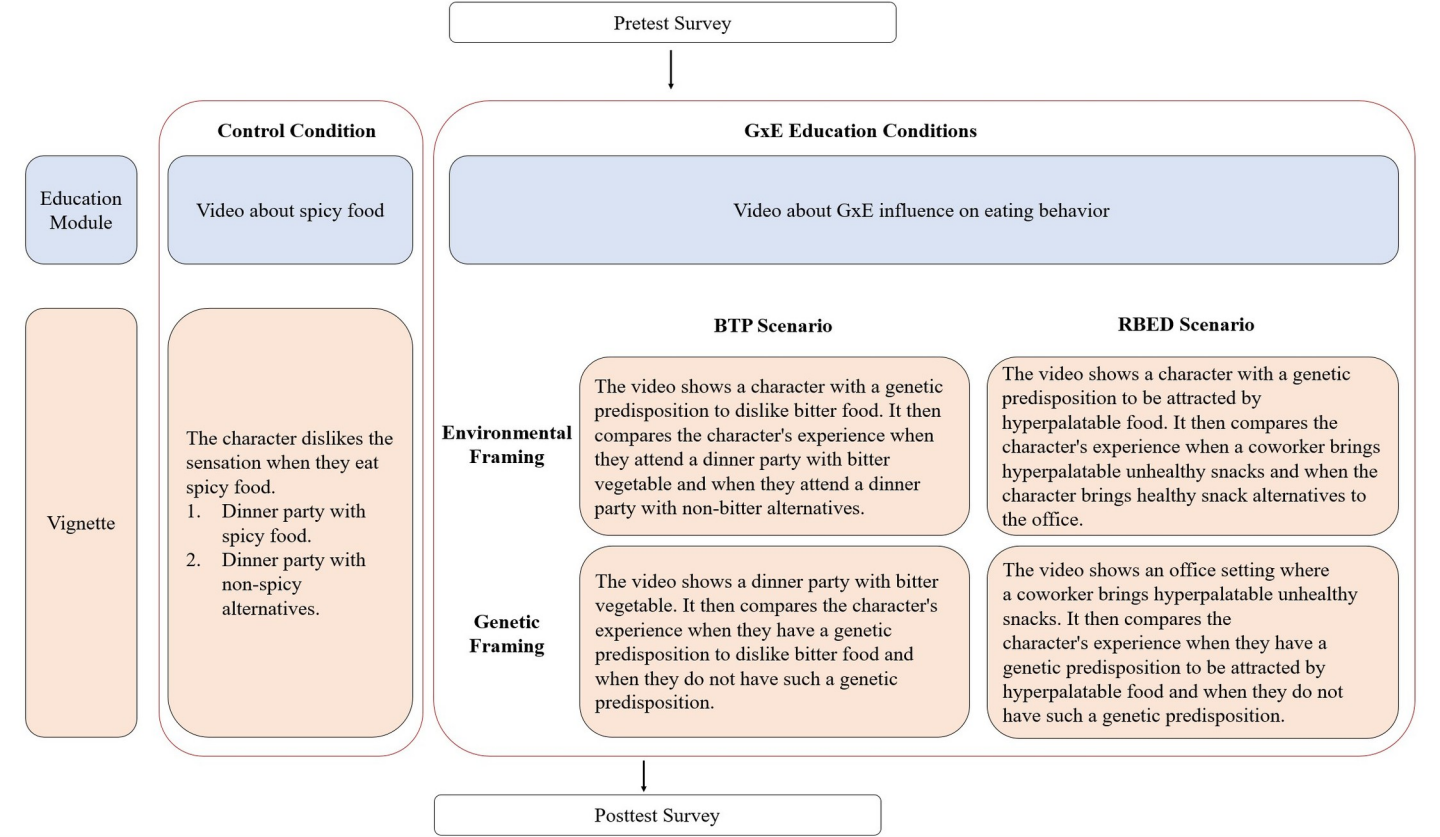
Study design and procedure elements.

The study was deemed IRB exempt through the National Institutes of Health Office of Human Subjects Research. Participants’ consent obtained by clicking a box indicating agreement in an online survey. The procedure and study design are shown in Fig 1. First, participants filled out an online survey that included questions about their eating behaviors and related attitudes. Next, participants were randomly assigned to one of the four GxE education vignette conditions or the control condition. In the GxE education conditions, participants first watched a GxE educational video introducing GxE concepts and explaining how the influence of genes depends on a person’s environment and vice versa. After that, participants watched one of the four narrative vignettes depicting how the character’s experience with eating hyperpalatable or bitter taste food (RBED vs. BTP scenario) is influenced by genetic or environmental variations (genetic vs. environmental framings). In the control condition, participants first watched an educational video about spicy food, and then watched a vignette video that demonstrated how one’s experience during a dinner party might unfold differently if the food spice level was different. The educational videos were created by the research team based on an earlier version in a previous study [10]. Links to all videos are available in S1 Appendix. Finally, participants completed knowledge checks and answered questions about empathy, weight bias, and behavior change motivation.

### Participants

To calculate the required sample size, we used a conservative effect size associated with between-group differences in the assessment of digital learning materials wherein Cohen’s *f* = .12 [53]. With a significance criterion of α = .05 and power = .80, the minimum sample size needed with this effect size was *N* = 673 for a fixed effects ANOVA with five groups. 826 participants were recruited via Amazon Mechanical Turk through CloudResearch. Participants were excluded if they discontinued the survey prior to watching videos (*n* = 29), did not agree to be honest or refrain from using outside sources to answer outside questions (*n* = 39), were unable to see or hear videos (*n* = 66), indicated there was a reason to exclude their data (*n* = 35), self-reported unrealistic height/weight (*n* = 3), or had poor quality answers to open-ended questions (*n* = 8). Our final sample consisted of 685 adults with a mean age of 39.93 (*SD* = 11.97). See Table 1 for additional demographics.

**Table 1:** Summary of demographic variables.

	*N*	Percentage %
**Gender**		
Male	340	50
Female	335	49
Other genders	10	1
**Race**		
White	537	78
Asian	40	6
Black	70	10
Other	38	6
**Marital status**		
Married	264	39
Single	247	36
Widowed/Divorced/Separated	86	12
Non-Marital Relationship	68	10
Other	13	3
**Sexuality**		
Bisexual	60	9
Gay/ Lesbian	25	4
Straight	579	85
Other	20	2
**Self-reported weight status**		
Overweight	362	53
Not Overweight	323	47
**Reward based eating drive trait**		
High	330	48
Low	355	52
**Bitter taste perception**		
High	300	44
Low	385	56

### Measures

#### Knowledge and belief

Knowledge recall was measured with 11 true-false items adapted from Martingano et al. [10] Some questions could be answered by recalling information from the educational videos, while others required higher-level generalization of the knowledge. Example items included “Genes have no influence on which flavors of food people enjoy” and “Some lifestyle choices affect people differently because they have different genes”. Participants were given 1 point for each correct answer and 0 points for an incorrect answer or a “don’t know” response (α = .71).

Knowledge application measure was created by the research team. Participants read about two characters who have different genetic predispositions in terms of how quickly they feel full while eating. In three scenarios, participants were asked to estimate the number of calories that the characters would consume on a scale from 0 to 2000 and explain the rationale for their calorie estimate in an open-ended question. The first scenario measured participants’ understanding of genetic influences on eating, the second measured understanding of environmental influences on eating, and the third measured understanding of an interactive influence between the two. Responses were coded by three researchers who achieved adequate intercoder reliability (Fleiss’ kappa ranging from .64 to .94). The number of correct responses, out of six, comprises the application knowledge score (α = .57). Questions and coding documents are available in S2 Appendix.

Genetic, environmental, and willpower causal beliefs about eating behavior were each measured by a single item on a scale from 1 = *Not at all* to 5 = *Extremely*. The three items are “How much do you think eating behavior is influenced by a person’s genes/the environment a person lives in/a person’s willpower?”

#### Empathy and weight bias

Empathetic concern was measured by asking participants to what extent they felt six empathic emotions towards the vignette characters (i.e., people whose attention is captured by delicious food nearby and people who experience green vegetables as extremely bitter for RBED and BTP scenarios, respectively) on a scale from 1 = *None at all* to 7 = *Extremely* [51]. The empathic emotions were sympathetic, warm, compassionate, softhearted, tender, and moved (α = .94).

Perspective taking was measured by a 7-item measure asking about participants’ tendency to adopt the psychological point of view of the vignette characters from 1 = *Not at all* to 7 = *Extremely* (adapted from the perspective taking subscale of the Interpersonal Reactivity Index [18]). An example item is “I try to put myself in their shoes.” (α = .83).

The measure of weight stereotyping was adapted from the Obese Persons Trait Survey [54]. Participants indicated how much each of 12 descriptors fit people who are overweight or obese on a scale from 1 = *Not at all* to 5 = *Extremely*. Principle component analysis showed a two-factor solution. The first factor included 6 items (i.e., kind, warm, optimistic, simple, honest, generous) of positive descriptors (α = .91). The second factor included 6 items (i.e., lazy, sloppy, self-indulgent, lacking self-discipline, clumsy, weak) of negative descriptors (α = .86).

Stigma towards people with higher weight was measured using the Anti-Fat Attitudes scale [55]. Participants were asked to indicate their agreement with 14 statements from 1 = *Strongly disagree* to 5 = *Strongly agree*. The scale included 3 subscales: dislike, fear, and willpower (α = .89, .83, .79, respectively).

#### Behavior change motivation

Participants’ confidence in their ability to control their diet and weight was measured using 4 items on a scale of 1 = *Not at all* to 5 = *Extremely*. The measure was adapted from Martingano et al. [10]. The items are “How confident are you in your ability to control your diet/weight?” and “How much do you believe that there are things you can do to control your diet/weight?” (α = .84).

Self-efficacy to maintain a healthy weight and eating pattern was measured by the Healthy Eating and Weight Self-Efficacy (HEWSE) Scale [56]. The scale includes 11 items with two factors, namely healthy eating (α = .88) and healthy weight (α = .93). Participants responded on a scale of 1 = *Not at all* to 5 = *Extremely*.

Intention to change diet in the next 6 months was measured using 6 items adapted from Martingano et al. [10] on a scale of 1 = *Not at all* to 5 = *Extremely*. Example items are “How likely is it that you will change your diet in the next 6 months?” and “I intend to make changes to my food environment in the next 6 months?” (α = .96).

#### Individual eating behavior traits

RBED trait was measured by two items created by the research team. Participants answered the two questions “To what extent are you more likely to notice and think about delicious foods in your environment?” and “To what extent do you eat even when you’re not hungry?” on a scale of 1 = *Not at all* to 5 = *Extremely* (α = .62). Principal component analysis showed that the two items loaded on a single factor. Therefore, we averaged the item scores and recoded it into a dichotomous variable. Participants who had an average score higher (lower) than 2.5 (scale midpoint) was categorized as high/low RBED.

BTP trait was measured by one item created by the research team. Participants answered the question “To what extent do you perceive foods like kale, brussels sprouts, or grapefruit as very bitter?” on a scale of 1 = *Not at all* to 5 = *Extremely*. The score was recoded into a dichotomous variable. Participants who had an average score higher (lower) than midpoint (2.5) was categorized as high/low BTP.

### Data analysis

Pearson correlations were examined to assess the relationships among outcome variables. Regarding the hypotheses, independent sample t-tests were conducted to test the effect of GxE education vignettes (across all frames and scenarios) compared to control (H1) and the effects of genetic framing and environmental framing compared to control individually (H2a and H3a). We use Bonferroni method to adjust for multiple tests. Specifically, we multiply the *p* values by the number of tests (*n* = 48) and compare with the α = 0.05 level to determine significance. After that, one-way Analyses of Covariance (ANCOVAs) were conducted to test the main effect of framing (H2b and H3b), controlling for the effect of scenario. Two-way ANCOVAs were used to test the matching effect between vignette versions and individual traits and beliefs (RQ1). Mediation analyses were conducted with PROCESS [57] to test the role of causal beliefs in mediating the effect of education conditions on downstream outcomes (RQ2). In each mediation model, genetic, environmental, and will power causal beliefs were entered as parallel mediators. Bootstraps of 5,000 samples were used to estimate indirect effect. For readers’ interest to determine how each of the five conditions was compared to each other, we conducted one-way Analyses of Variance (ANOVA) followed by post-hoc analysis with Tukey’s test to compare all conditions on the outcomes. The results are shown in S3 Table in S1 File and not discussed in the main text.

## Results

### Effect of GxE educational vignettes

Results showed that participants who received GxE education (including both participants in genetic framing condition and those in environmental framing condition) had higher GxE recall (*t*(683) = 11.20, *p* < .001), higher genetic causal beliefs (*t*(683) = 7.12, *p* < .001) and higher empathetic concern (*t*(683) = 3.53, *p* < .001) compared to the control condition. There were no significant differences between GxE education conditions and the control condition on GxE knowledge application, environmental and willpower causal beliefs, perspective taking, weight bias outcomes, or any of the behavior change motivation outcomes (see Table 2 and S9-S12 Figs in S2 File). H1 was partially supported.

**Table 2:** Variable means by group and T-tests comparing education conditions with control condition.

	Environ Framing (*N* = 276)^1^	Gene Framing (*N* = 269)^2^	Education [Both frames] (*N* = 545)^3^	Control (*N* = 140)^4^	Environ vs. Control		Gene vs. Control		Education vs. Control	
	*M*(*SD*)	*M*(*SD*)	*M*(*SD*)	*M*(*SD*)	*t*	*p*	*t*	*p*	*t*	*p*
**Knowledge and Belief Outcomes**										
Knowledge Recall (range 0–11)	9.46(1.54)	9.69(1.45)	9.57(1.50)	7.16(2.43)	10.19	**< .001**	11.31	**< .001**	11.20	**< .001**
Knowledge Application (range 0–6)	4.92(1.15)	4.83(1.19)	4.88(1.17)	4.91(1.23)	0.14	.89	-0.59	.55	-0.24	.81
Causal Beliefs (range 1–5)										
Genetic	3.41(0.84)	3.46(0.78)	3.44(0.81)	2.89(0.83)	6.04	**< .001**	6.92	**< .001**	7.12	**< .001**
Environment	3.89(0.76)	3.78(0.75)	3.84(0.75)	3.84(0.85)	0.59	.55	-0.81	.42	-0.11	.91
Willpower	3.31(0.92)	3.31(0.94)	3.31(0.93)	3.51(0.90)	-2.14	.03^4^	-2.13	.03^4^	-2.34	.02^4^
**Empathy Outcomes**										
Empathetic Concern (range 1–7)	4.41(1.57)	4.38(1.44)	4.40(1.51)	3.89(1.54)	3.20	**.001**	3.20	**.001**	3.53	**< .001**
Perspective Taking (range 1–7)	5.12(1.12)	5.16(1.16)	5.14(1.14)	5.12(1.04)	0.01	1.00	0.30	.76	0.17	.87
**Weight Bias Outcomes**										
Weight Stereotype (range 1–5)										
Negative	2.13(0.85)	2.18(0.80)	2.15(0.82)	2.17(0.85)	-0.46	.65	0.10	.92	-0.21	.84
Positive	3.06(0.79)	3.10(0.69)	3.08(0.74)	3.01(0.74)	0.63	.53	1.24	.22	1.01	.31
Weight Stigma (range 1–5)										
Dislike	1.82(0.83)	1.79(0.81)	1.80(0.82)	1.83(0.84)	-0.15	.88	-0.49	.63	-0.35	.73
Fear	3.21(1.18)	3.07(1.18)	3.14(1.18)	3.07(1.23)	1.14	.25	0.04	.97	0.66	.51
Willpower	2.85(0.89)	2.87(0.87)	2.86(0.88)	2.98(0.89)	-1.39	.17	-1.15	.25	-1.40	.16
**Behavior Change Motivation Outcomes**										
Confidence (range 1–5)	3.66(0.86)	3.57(0.83)	3.62(0.85)	3.73(0.78)	-0.77	.44	-1.80	.07	-1.38	.17
Self-Efficacy (range 1–5)										
Healthy Eating	4.03(0.73)	4.00(0.72)	4.02(0.73)	4.05(0.69)	-0.29	.79	-0.70	.48	-0.52	.60
Healthy Weight	3.63(1.04)	3.60(1.04)	3.61(1.03)	3.66(1.05)	-0.28	.78	-0.55	.58	-0.46	.65
Intention (range 1–5)	3.16(1.13)	3.06(1.13)	3.11(1.13)	2.98(1.15)	1.47	.14	0.62	.54	1.16	.25

*Notes*.

^1^ *N* = 274

^2^ *N* = 265

^3^ *N* = 539, and

^4^ *N* = 238 for numeric and textual knowledge application. ^4^ Not significant with Bonferroni adjustment.

### Framing effects

Genetic framing resulted in higher empathetic concern compared to the control condition (*t*(407) = 3.202, *p* = .001), while perspective taking and weight bias outcomes were not significantly different between the two conditions. H3a was partially supported. Behavior change motivation outcomes were not significantly different between environmental framing and the control condition (see Table 2). H3b was not supported.

There was no significant difference between the genetic and environmental framing conditions on any outcomes (see S4 Table in S1 File). H2b and H3b were not supported.

### Tailoring to individual traits

#### RBED trait as a moderator

**Fig 2:**
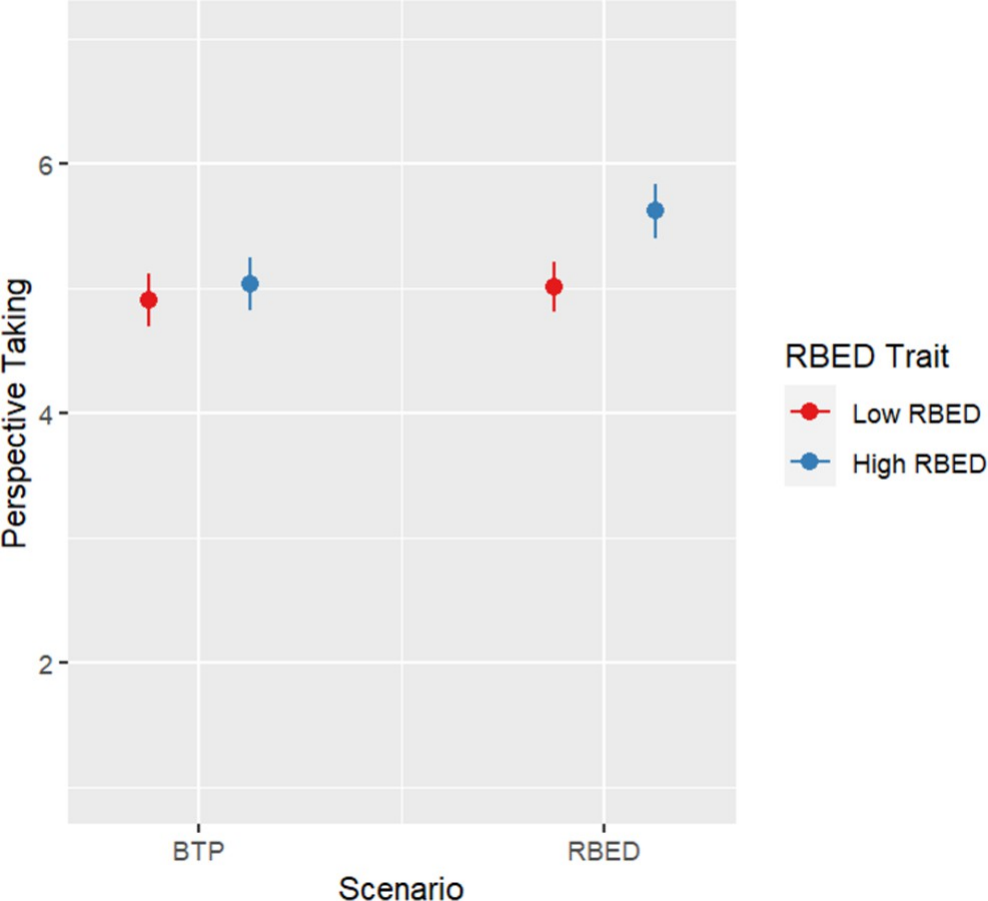
Plot for perspective-taking results by condition.

Table 3 shows results of ANCOVAs testing the Framing-RBED trait interactions and Scenario-RBED trait interactions on the outcomes. There was a significant interaction between Scenario and RBED trait on perspective taking (*F*(1, 540) = 6.256. *p* = .013). Individuals with high RBED trait reported significantly more perspective taking in the RBED scenario than in the BTP scenario (*F*(1, 261) = 19.935. *p* < .001) while there was no significant difference between scenarios for individuals with low RBED trait (Fig 2).

**Table 3:** Two-way ANCOVAs testing framing-RBED trait and scenario-RBED trait interactions.

	Model 1			Model 2		
	Framing	RBED Trait	Framing* RBED Trait	Scenario	RBED Trait	Scenario* RBED Trait
**Knowledge and Belief Outcomes**						
Knowledge Recall (range 0–11)	3.14	0.11	0.00	16.40***	0.11	2.47
Knowledge Application (range 0–6)	0.86	0.09	0.78	0.35	0.09	0.00
Causal Beliefs (range 1–5)						
Genetic	0.59	9.74**	0.14	1.72	9.74**	0.11
Environment	3.11	1.78	0.05	0.65	1.78	0.10
Willpower	0.00	0.05	0.15	0.38	0.05	0.30
**Empathy Outcomes**						
Empathetic Concern (range 1–7)	0.06	5.52*	0.62	12.29***	5.53*	1.83
Perspective Taking (range 1–7)	0.10	14.75***	0.06	10.94**	14.92***	6.26*
**Weight Bias Outcomes**						
Weight Stereotype (range 1–5)						
Negative	0.48	0.00	0.48	0.03	0.00	1.99
Positive	0.39	2.96	4.75*	1.10	2.94	0.83
Weight Stigma (range 1–5)						
Dislike	0.15	3.69	0.07	2.43	3.69	0.01
Fear	1.83	8.90**	0.06	0.41	8.90**	0.26
Willpower	0.10	1.63	1.37	1.78	1.63	0.36
**Behavior Change Motivation Outcomes**						
Confidence (range 1–5)	1.52	39.18***	3.59	0.08	39.02	1.39
Self Efficacy (range 1–5)						
Healthy Eating	0.27	20.69***	5.59*	0.12	20.53***	1.320
Healthy Weight	0.12	56.81***	4.46*	0.00	56.36***	0.14
Intention (range 1–5)	1.21	80.59***	0.07	0.25	80.59***	0.05

*Notes*.

*p < .05

** *p* < .01

*** *p* < .001.

The table shows *F* values. Model 1 controlled for scenario. Model 2 controlled for framing.

**Fig 3:**
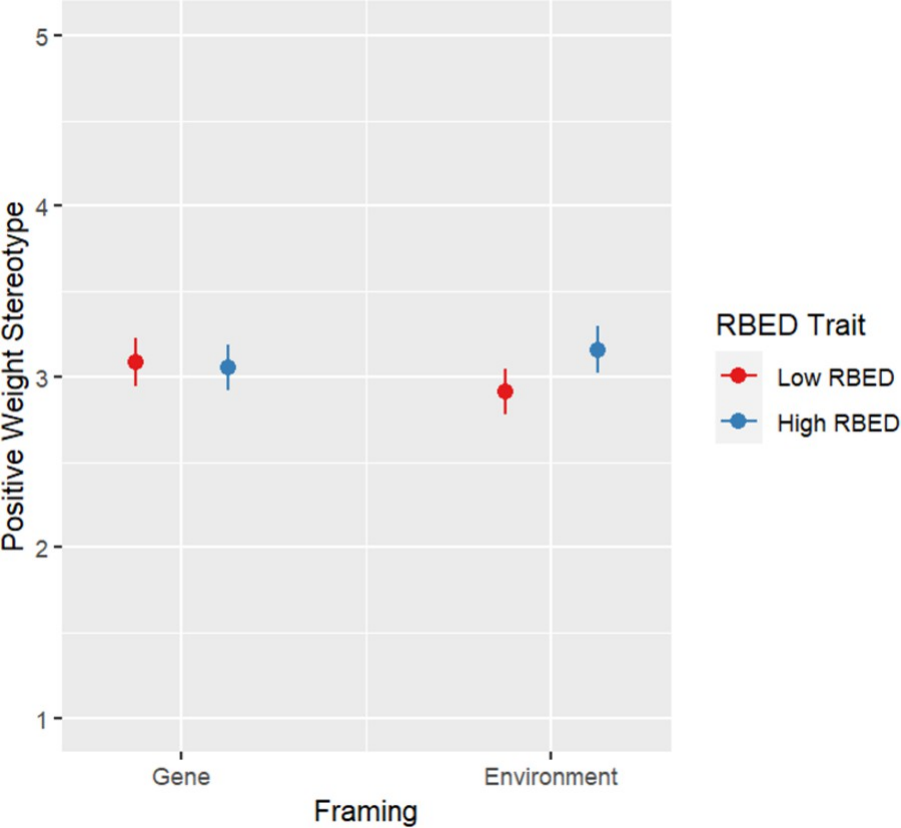
Plot for positive weight stereotype by condition.

There was a significant Framing-RBED trait interaction on positive stereotyping of overweight (*F*(1, 540) = 4.751, *p* = .030). Individuals with low RBED trait reported significantly higher positive weight stereotypes in the genetic framing condition than in the environment framing condition (*F*(1, 278) = 4.193. *p* = .042) while there was no significant difference between framing types for individuals with high RBED trait (Fig 3).

**Fig 4:**
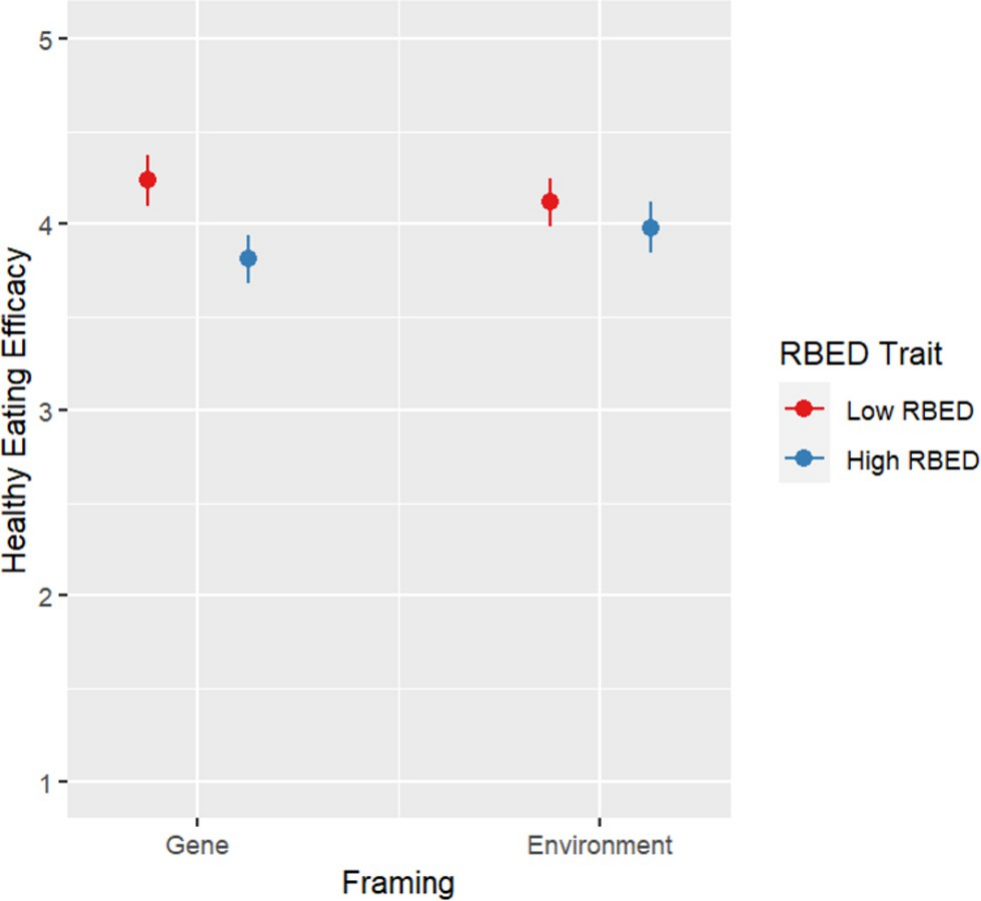
Plot for health eating efficacy results by condition.

**Fig 5:**
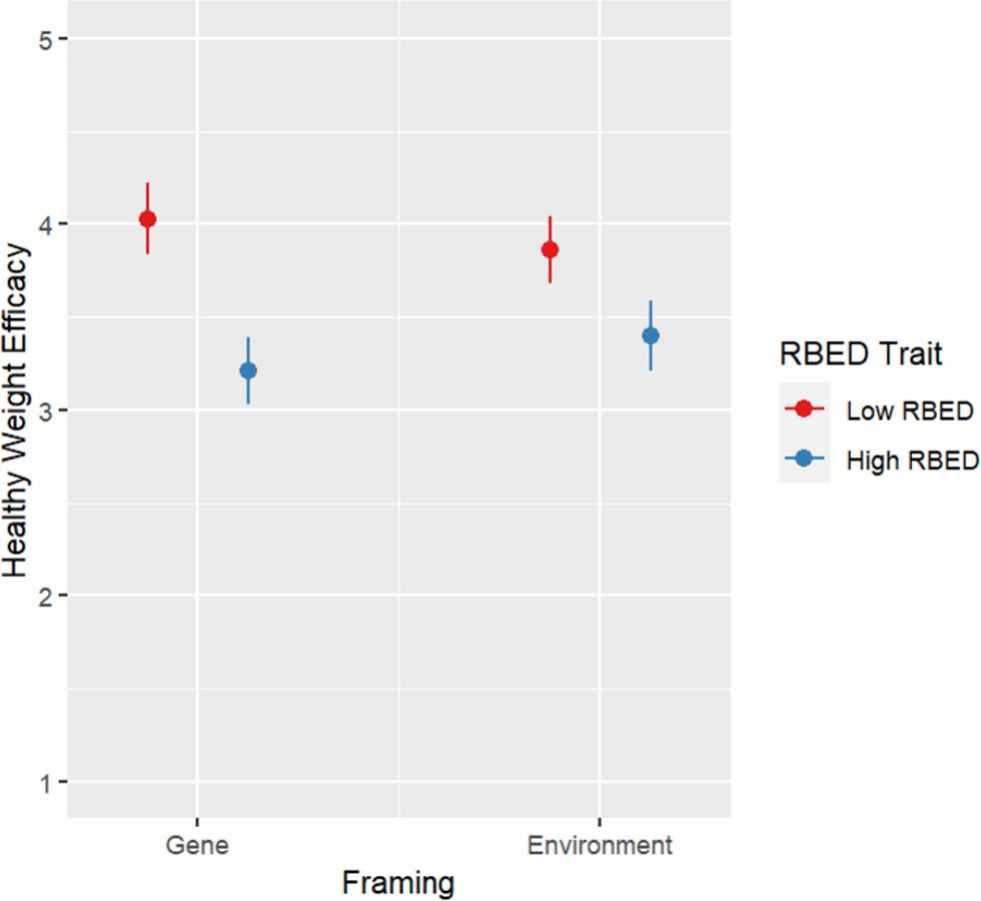
Plot for health weight efficacy results by condition.

There was a significant interaction Framing-RBED trait interaction on healthy eating efficacy (*F*(1, 540) = 5.590, *p* = .018) and healthy weight efficacy (*F*(1, 540) = 4.457, *p* = .035). Simple effect analyses did not show any significant differences (Figs 4 and 5).

#### BTP as a moderator

S5 Table in S1 File shows results of ANCOVAs testing the Framing-BTP trait interactions and Scenario-BTP trait interactions on the outcomes. There were no significant interactions in either case.

### Mediation models

Zero-order correlations (Table 4) show that knowledge recall and application were rarely correlated with downstream outcome variables (exceptions include positive stereotyping and the fear and willpower subscales of the weight stigma measure). Causal beliefs, on the other hand, were highly correlated, either positively or negatively, with most outcome variables, which suggests potential mediation or indirect effects of causal beliefs on key outcomes.

**Table 4:** Pearson correlations among outcomes.

Variable	1	2	3	4	5	6	7	8	9	10	11	12	13	14	15	16
1. Knowledge Recall	-															
2. Knowledge Application	0.22***															
3. Genetic Causal Belief	0.21***	-0.03	-													
4. Environmental Causal Belief	0.06	0.04	0.32***	-												
5. Willpower Causal Belief	-0.09*	0.01	-0.08	0.17***	-											
6. Empathetic Concern	0.06	-0.01	0.28***	0.11**	-0.15***	-										
7. Perspective Taking	0.01	0.04	0.20***	0.09*	-0.15***	0.54***	-									
8. Negative Weight Stereotype	0.00	0.03	-0.13**	0.04	0.50***	-0.18***	-0.24***	-								
9. Positive Weight Stereotype	-0.08*	-0.13***	0.25***	0.18***	0.03	0.34***	0.24***	-0.13***	-							
10. Dislike (Weight Stigma)	-0.02	0.04	-0.24***	-0.12**	0.21***	-0.24***	-0.29***	0.60***	-0.30***	-						
11. Fear (Weight Stigma)	0.03	0.17***	-0.03	0.01	0.19***	-0.04	-0.07	0.30***	-0.05	0.27***	-					
12. Willpower (Weight Stigma)	-0.02	0.12**	-0.35***	-0.11*	0.48***	-0.24***	-0.28***	0.68***	-0.20***	0.54***	0.32***	-				
13. Confidence	0.00	0.01	-0.09*	0.16***	0.42***	-0.07	-0.01	0.25***	0.03	0.16***	-0.06	0.34***	-			
14. Healthy Eating Efficacy	0.01	0.03	-0.01	0.10*	0.18***	0.09*	0.10**	0.00	0.12**	-0.04	-0.08*	0.09*	0.52***	-		
15. Healthy Weight Efficacy	-0.01	-0.03	-0.05	0.07	0.24***	0.03	0.07	0.16***	0.06	0.13**	-0.17**	0.22***	0.71***	0.56***	-	
16. Intention	-0.02	0.02	0.21***	0.11*	0.13***	0.19***	0.14***	0.03	0.24***	-0.09*	0.33***	-0.02	-0.10*	0.01	-0.18***	-

*Notes*.

*p < .05

**p < .01

***p < .001

Mediation models (see S6-S8 Tables in S1 File) for the effects of education conditions versus control reveal a consistent indirect effect of genetic causal beliefs as a mediator of the effects on several dependent variables. Specifically, being in one of the G*E education conditions increased participants’ beliefs in genetic causes of eating behavior, which in turn led to increased empathy, increased perspective taking, reduced negative stereotyping, increased positive stereotyping, reduced dislike and willpower weight stigma, decreased dietary confidence, and greater intentions to change dietary behavior. There was no significant indirect effect of genetic causal beliefs for the fear subscale of the weight stigma measure and self-efficacy for healthy eating and weight. Being in one of the G*E education conditions also reduced participants’ beliefs in willpower causes of eating behavior, which in turn led to increased empathy, increased perspective taking, reduced negative stereotyping, reduced dislike, fear, and willpower weight stigma, reduced confidence and self-efficacy of healthy eating and weight, and reduced intentions to change dietary behavior. Finally, there were no indirect effects through environmental causal beliefs on the downstream outcomes.

## Discussion

The current study indicates that our GxE educational intervention we developed successfully improved recall knowledge and altered genetical causal beliefs. However, the intervention rarely directly impacted downstream outcomes related to dietary and weight management behavior motivation or to empathy and stigma concerning eating tendencies and weight. Although the relationships between GxE education and downstream outcomes were theory-based, the overall pattern of results was mixed, even when matching individuals’ eating behavior tendencies with frames and vignette scenarios.

There could be several explanations for these findings. First, application knowledge and environmental causal beliefs did not differ by condition, possibly due to ceiling effects. Environmental causal beliefs regarding eating behavior are generally high as compared with genetic beliefs [27] and were also relatively high in this sample. The knowledge application questions appear to have been too easy to successfully differentiate individuals with different knowledge levels; more than 65% participants correctly answered 5 or all 6 questions. Future research should explore alternative instruments to assess GxE application knowledge.

The only downstream outcome improved by educational vignettes overall was empathy toward people with specific eating behavior traits. The lack of intervention effect on other downstream targets is likely related to the fact that these concepts were not explicitly mentioned in the educational material. Instead, changes in causal beliefs and reductions in blame were proposed as routes to influence these outcomes. This study found that GxE education influenced genetic and willpower causal beliefs which, in turn influenced the majority of empathy, stigma, and behavior motivation outcomes. The findings suggest that altering genetic and willpower causal beliefs is a critical precondition for influencing downstream outcomes. Future GxE educational interventions should focus on strategies to increase genetic causal beliefs in order to reduce stigma beliefs while considering the potential negative consequences on behavior change motivations due to weakened willpower causal beliefs and heightened belief in unmodifiable genetic factors. Although reduced willpower causal beliefs are associated with less stigma, they may also reduce health behavior motivation. It may therefore be useful to consider how we might replace “willpower” causal beliefs for eating behavior with a more helpful and accurate set of beliefs that capture how healthy behaviors can be influential on health outcomes within the context of genetic predisposition. In the current study, this was represented by environmental framing. Although here environmental causal beliefs were not influenced by GxE education, we should not disregard the role of environmental causal beliefs. Rather, in future studies we may need to consider better ways to describe in interventions and measure individuals’ understanding regarding the multiple facets of environmental causes such as home environment, social support and network, available foods and health resources. Additionally, exploring how causal beliefs about these environmental facets influence stigma and health behavior motivations is crucial.

The other primary assessment was of the potential for differential effects of different GxE education interventions based on individuals’ eating behavior traits. Per the literature on tailoring effects [39], individuals should be more likely to take the perspective of the character when the character has a similar eating tendency to them. However, this perspective-taking boost was only found for RBED trait and did not translate into other empathy or stigma-related changes.

Genetic framing may reduce stigma toward individuals with relatively stigmatized eating tendencies, particularly among those who do not have direct experience with a given maladaptive eating behavior. In this study, low RBED trait individuals exhibited more positive stereotyping of people with obesity in the genetic framing condition than the environmental framing while there was no significant difference between framing types for high RBED trait individuals. The finding shows that experiential frameworks may be helpful in improving attitudes among those who lack experience with RBED and who may make assumptions about why others are excessively drawn to hyperpalatable food in the environment [58]. Future GxE education experiences may be strengthened to further reduce stigma related to eating behavior and weight by leveraging this finding. A first step may be the use of a genetic (as opposed to environmental) framing for GxE education.

Environmental framing, on the other hand, may benefit individuals with high RBED trait. Though simple effects were not statistically significant, significant interaction patterns suggest that dietary and weight self-efficacy may be stronger for high RBED individuals whose educational content focuses on environmental influence compared to nonmodifiable genetic influences. This is consistent with previous literature suggesting the potential for genetic attributions or GxE information to elicit deterministic responses among those most vulnerable to negative health outcomes [26, 46]. Such patterns, if replicated, suggest a potential benefit of adopting differential frames based on whether or not individuals exhibit the trait or disease of focus.

### Limitations and future directions

The results provide preliminary evidence that targeting specific types of GxE education interventions to individuals with certain traits may enhance the desired intervention benefits. However, the effect sizes across the outcomes were small, meaning that the intervention impact in the real world would likely be limited, although these small effects are comparable to other similar video-based interventions [10, 59]. The video vignettes may be used in combination with other educational elements in future interventions to create a larger impact.

In addition, although the vignettes were intended to be experiential, they were presented in a video format. The video format, combined with a second-person (i.e., “you”) narrative, allowed individuals to take the character’s perspective but lacked immersiveness. Research suggests that embodying stigmatized group members’ experiences can reduce stigma through increased empathy [60–62]. Therefore, intervention effects on empathy and stigma may be more potent with a more immersive experience. Future studies should explore delivering GxE education interventions using technologies, such as virtual reality.

Furthermore, the knowledge check and knowledge application measures used in this study were pilot tested, however they were not validated, and the reliability of the knowledge application measure was low. This may partially explain why the results did not show a significant impact of the interventions on knowledge application. A validated scale to measure GxE knowledge application is needed, as the ability to apply GxE knowledge to various health contexts is crucial for individuals to make positive health decisions in the future.

Lastly, we did not preregister all of the reported analyses and did not measure several potential mechanisms for effects. Future research should replicate these findings and to further explore the mechanisms of any framing or tailoring effects that might emerge to better inform GxE intervention targeting practices.

## Conclusions

In conclusion, this study designed and tested the efficacy of an experiential, vignette based GxE education intervention in the context of eating behavior traits. The results reveal that causal attributions, particularly to genetics and willpower, are key factors that drive downstream stigma and eating behavior-related outcomes, and thus may be promising targets for future interventions. Although anticipated framing effects were not found, matching frames with individuals’ eating behavior traits, particularly RBED, revealed intervention effects that were not observed when evaluating effects among the general sample. This indicates that while delivering GxE education interventions to the general public improves knowledge and causal beliefs, tailoring GxE education interventions to individual traits may lead to more meaningful downstream outcomes.

## Supporting information

S1 Appendix Video links. (DOCX)

S2 Appendix Knowledge application measure and coding document. (DOCX)

S1 File S3-S8 Tables. Omnibus test, ANCOVAs, and mediation analysis results. (DOCX)

S2 File S9-S13 Figs. Means of outcomes comparing education and control conditions. (DOCX)

S1 Checklist *PLOS ONE* clinical studies checklist. (DOCX)

## References

[1] Hunter DJ. Gene–environment interactions in human diseases. Nat Rev Genet. 2005;6(4):287–98. doi: 10.1038/nrg1578 15803198

[2] Lewis CM, Vassos E. Polygenic risk scores: from research tools to clinical instruments. Genome Med. 2020;12(1):44. doi: 10.1186/s13073-020-00742-5 32423490 PMC7236300

[3] Wallingford CK, Kovilpillai H, Jacobs C, Turbitt E, Primiero CA, Young MA, et al. Models of communication for polygenic scores and associated psychosocial and behavioral effects on recipients: a systematic review. Genet Med. 2022;25(1):1–11. doi: 10.1016/j.gim.2022.09.008 36322150

[4] Grishkevich V, Yanai I. The genomic determinants of genotype× environment interactions in gene expression. Trends Genet. 2013;29(8):479–87. 23769209 10.1016/j.tig.2013.05.006

[5] Condit CM, Shen L. Public understanding of risks from gene-environment interaction in common diseases: implications for public communications. Public Health Genomics. 2011;14(2):115–24. doi: 10.1159/000314915 20714109 PMC3214934

[6] Zang J, Hammann M. Promoting students’ understanding of gene-environment interaction in genetics education. In: Korfiatis K, Grace M, editors. Current research in biology education: Selected papers from the ERIDOB community. Springer International Publishing; 2022. p. 167–80.

[7] Boerwinkel DJ, Yarden A, Waarlo AJ. Reaching a consensus on the definition of genetic literacy that is required from a twenty-first-century citizen. Sci Educ. 2017;26:1087–114.

[8] Aspinwall LG, Drummond DM, Stump TK, Kohlmann WK, Leachman SA. Interactive beliefs about genes and behavior predict improved sun protection following melanoma genetic counseling. Ann Behav Med. 2022;56(8):816–29. doi: 10.1093/abm/kaab117 35179177 PMC9345182

[9] Carver RB, Castéra J, Gericke N, Evangelista NAM, El-Hani CN. Young adults’ belief in genetic determinism, and knowledge and attitudes towards modern genetics and genomics: the PUGGS questionnaire. PloS One. 2017;12(1):e0169808. doi: 10.1371/journal.pone.0169808 28114357 PMC5256916

[10] Martingano AJ, Telaak SH, Schopp EM, Fortney C, Dolwick AP, Carnell S, et al. Using educational videos and perspective-taking to communicate gene-by-environment interaction concepts about eating behavior: effects on empathy and weight stigma. J Nutr Educ Behav. 2023;55(1):55–67. doi: 10.1016/j.jneb.2022.09.005 36621267 PMC9833839

[11] Ke S, Lai J, Sun T, Yang MMH, Wang JCC, Austin J. Healthy young minds: the effects of a 1-hour classroom workshop on mental illness stigma in high school students. Community Ment Health J. 2015 Apr;51(3):329–37. doi: 10.1007/s10597-014-9763-2 25017811 PMC4318697

[12] Cheng Y, Condit C, Flannery D. Depiction of gene-environment relationships in online medical recommendations. Genet Med. 2008;10(6):450–6. doi: 10.1097/gim.0b013e31817701a8 18496221

[13] Horwitz AV. Media portrayals and health inequalities: a case study of characterizations of gene x environment interactions. J Gerontol B Psychol Sci Soc Sci. 2005;60:48–52. doi: 10.1093/geronb/60.special_issue_2.s48 16251590

[14] Condit CM, Gronnvoll M, Landau J, Shen L, Wright L, Harris TM. Believing in both genetic determinism and behavioral action: a materialist framework and implications. Public Underst Sci. 2009;18(6):730–46.

[15] Kaphingst KA, Persky S, McCall C, Lachance C, Beall AC, Blascovich J. Testing communication strategies to convey genomic concepts using virtual reality technology. J Health Commun. 2009 Oct 1;14(4):384–99. doi: 10.1080/10810730902873927 19466649 PMC2767374

[16] Kaphingst KA, Persky S, McCall C, Lachance C, Loewenstein J, Beall AC, et al. Testing the effects of educational strategies on comprehension of a genomic concept using virtual reality technology. Patient Educ Couns. 2009 Aug 11;77(2):224–30. doi: 10.1016/j.pec.2009.03.029 19409749 PMC2794484

[17] Kral TV, Faith MS, Pietrobelli A, Allison DB, Baskin ML. Assessment of eating and weight-related problems in children. In: Allison DB, Baskin ML, editors. Handbook of assessment methods for eating behaviors and weight-related problems. Sage; 2009. p. 447–79.

[18] Davison KK, Francis LA, Birch LL. Reexamining obesigenic families: parents’ obesity‐related behaviors predict girls’ change in BMI. Obes Res. 2005;13(11):1980–90. doi: 10.1038/oby.2005.243 16339130 PMC2530936

[19] Khera AV, Chaffin M, Wade KH, Zahid S, Brancale J, Xia R, et al. Polygenic prediction of weight and obesity trajectories from birth to adulthood. Cell. 2019 May 2;177(3):587–96. doi: 10.1016/j.cell.2019.03.028 31002795 PMC6661115

[20] Grimm ER, Steinle NI. Genetics of eating behavior: established and emerging concepts. Nutr Rev. 2011;69(1):52–60. doi: 10.1111/j.1753-4887.2010.00361.x 21198635 PMC3052625

[21] Cook TM, Wang J. Causation beliefs and stigma against depression: results from a population-based study. J Affect Disord. 2011;133(1–2):86–92. doi: 10.1016/j.jad.2011.03.030 21489636

[22] Pearl RL, Lebowitz MS. Beyond personal responsibility: effects of causal attributions for overweight and obesity on weight-related beliefs, stigma, and policy support. Psychol Health. 2014 Jan 1;29(10):1176–91. doi: 10.1080/08870446.2014.916807 24754230

[23] Langford AT, Solid CA, Gann LC, Rabinowitz EP, Williams SK, Seixas AA. Beliefs about the causes of hypertension and associations with pro-health behaviors. Health Psychol. 2018;37(12):1092–101. doi: 10.1037/hea0000687 30307273 PMC10148973

[24] Hilbert A. Weight stigma reduction and genetic determinism. PloS One. 2016;11(9):e0162993. doi: 10.1371/journal.pone.0162993 27631384 PMC5025056

[25] Jeong SH. Effects of news about genetics and obesity on controllability attribution and helping behavior. Health Commun. 2007;22(3):221–8. doi: 10.1080/10410230701626877 17967144

[26] Persky S, Goldring MR, El-Toukhy S, Ferrer RA, Hollister B. Parental defensiveness about multifactorial genomic and environmental causes of children’s obesity risk. Child Obes. 2019;15(5):289–97. doi: 10.1089/chi.2018.0315 30946599 PMC6590722

[27] Persky S, Bouhlal S, Goldring MR, McBride CM. Beliefs about genetic influences on eating behaviors: characteristics and associations with weight management confidence. Eat Behav. 2017 Feb;26:93–8. doi: 10.1016/j.eatbeh.2017.02.003 28199907 PMC5545160

[28] Persky S, Yaremych HE. Parents’ genetic attributions for children’s eating behaviors: relationships with beliefs, emotions, and food choice behavior. Appetite. 2020 Oct;155:104824. doi: 10.1016/j.appet.2020.104824 32781082 PMC8121139

[29] Wicks RH. Message framing and constructing meaning: an emerging paradigm in mass communication research. Ann Int Commun Assoc. 2005;29(1):335–62.

[30] Li N, Su LYF. Message framing and climate change communication: a meta-analytical review. J Appl Commun. 2018;102(3):1c.

[31] Rosenstock IM. Historical origins of the health belief model. Health Educ Monogr. 1974;2(4):328–35. 10.1177/109019817800600406 299611

[32] Chang C. Behavioral recommendations in health research news as cues to action: self-relevancy and self-efficacy processes. J Health Commun. 2016;21(8):954–68. doi: 10.1080/10810730.2016.1204377 27442057

[33] Beauchamp MR, Rhodes RE, Kreutzer C, Rupert JL. Experiential versus genetic accounts of inactivity: implications for inactive individuals’ self-efficacy beliefs and intentions to exercise. Behav Med. 2011;37(1):8–14. doi: 10.1080/08964289.2010.540263 21347905

[34] Weiner B. On sin versus sickness: a theory of perceived responsibility and social motivation. Am Psychol. 1993;48(9):957–65. 8214914 10.1037//0003-066x.48.9.957

[35] Easter MM. “Not all my fault”: genetics, stigma, and personal responsibility for women with eating disorders. Soc Sci Med. 2012;75(8):1408–16. doi: 10.1016/j.socscimed.2012.05.042 22819736 PMC3495131

[36] Kvaale EP, Gottdiener WH, Haslam N. Biogenetic explanations and stigma: a meta-analytic review of associations among laypeople. Soc Sci Med. 2013;96:95–103. doi: 10.1016/j.socscimed.2013.07.017 24034956

[37] Hilbert A, Rief W, Braehler E. Stigmatizing attitudes toward obesity in a representative population‐based sample. Obesity. 2008;16(7):1529–34. doi: 10.1038/oby.2008.263 18464749

[38] Persky S, Eccleston CP. Medical student bias and care recommendations for an obese versus non-obese virtual patient. Int J Obes. 2011 May 1;35(5):728–35. doi: 10.1038/ijo.2010.173 20820169 PMC3000449

[39] Kreuter MW, Wray RJ. Tailored and targeted health communication: strategies for enhancing information relevance. Am J Health Behav. 2003;27(1):S227–32. doi: 10.5993/ajhb.27.1.s3.6 14672383

[40] Persky S, Ferrer RA, Klein WM. Genomic information may inhibit weight-related behavior change inclinations among individuals in a fear state. Ann Behav Med. 2016;50(3):452–9. doi: 10.1007/s12160-016-9771-2 26850762 PMC4867291

[41] McHugh J, Suggs LS. Online tailored weight management in the worksite: does it make a difference in biennial health risk assessment data? J Health Commun. 2012 Jan 1;17(3):278–93. doi: 10.1080/10810730.2011.626496 22188131

[42] Epel ES, Tomiyama AJ, Mason AE, Laraia BA, Hartman W, Ready K, et al. The reward-based eating drive scale: a self-report index of reward-based eating. PloS One. 2014;9(6):e101350. doi: 10.1371/journal.pone.0101350 24979216 PMC4076308

[43] Dolwick AP, Persky S. Parental reward-based eating drive predicts parents’ feeding behaviors and children’s ultra-processed food intake. Appetite. 2021;164:105241. doi: 10.1016/j.appet.2021.105241 33839147 PMC8529800

[44] Mason AE, Vainik U, Acree M, Tomiyama AJ, Dagher A, Epel ES, et al. Improving assessment of the spectrum of reward-related eating: the RED-13. Front Psychol. 2017;8:795. doi: 10.3389/fpsyg.2017.00795 28611698 PMC5447741

[45] Bell KI, Tepper BJ. Short-term vegetable intake by young children classified by 6-n-propylthoiuracil bitter-taste phenotype. Am J Clin Nutr. 2006;84(1):245–51. doi: 10.1093/ajcn/84.1.245 16825702

[46] Gbenro MO, Martingano AJ, Persky S. Exploring the impact of genetic beliefs about specific eating behaviors on dietary self-efficacy. J Behav Med. 2022 Jun;45(3):497–502. doi: 10.1007/s10865-022-00290-w 35103881 PMC9995156

[47] Kim UK, Drayna D. Genetics of individual differences in bitter taste perception: lessons from the PTC gene. Clin Genet. 2005 Apr 1;67(4):275–80. doi: 10.1111/j.1399-0004.2004.00361.x 15733260

[48] Ravuri S, Martingano A, Persky S. Evaluating eating behavior traits of virtual targets: attitudes and empathy. under review; doi: 10.1016/j.eatbeh.2023.101808 37699308 PMC11301783

[49] Avraamidou L, Osborne J. The role of narrative in communicating science. Int J Sci Educ. 2009;31(12):1683–707.

[50] Arya DJ, Maul A. The role of the scientific discovery narrative in middle school science education: An experimental study. J Educ Psychol. 2012;104(4):1022–32.

[51] Batson CD. The altruism question: toward a social-psychological answer. Psychology Press; 2014.

[52] Todd AR, Galinsky AD. Perspective‐taking as a strategy for improving intergroup relations: evidence, mechanisms, and qualifications. Soc Personal Psychol Compass. 2014;8(7):374–87.

[53] Chen HTM, Thomas M. Effects of lecture video styles on engagement and learning. Education Tech Research Dev. 2020; 68, 2147–64.

[54] Puhl RM, Schwartz MB, Brownell KD. Impact of perceived consensus on stereotypes about obese people: a new approach for reducing bias. Health Psychol. 2005;24(5):517–25. doi: 10.1037/0278-6133.24.5.517 16162046

[55] Crandall CS. Prejudice against fat people: ideology and self-interest. J Pers Soc Psychol. 1994;66(5):882–94. doi: 10.1037//0022-3514.66.5.882 8014833

[56] Wilson-Barlow L, Hollins TR, Clopton JR. Construction and validation of the healthy eating and weight self-efficacy (HEWSE) scale. Eat Behav. 2014;15(3):490–2. doi: 10.1016/j.eatbeh.2014.06.004 25064304

[57] Hayes AF. Introduction to mediation, moderation, and conditional process analysis: a regression-based approach. Guilford Publications; 2017.

[58] Barker ME, Tandy M, Stookey JD. How are consumers of low-fat and high-fat diets perceived by those with lower and higher fat intake? Appetite. 1999;33(3):309–17. doi: 10.1006/appe.1999.0248 10625524

[59] Hopfer S. Effects of a narrative HPV vaccination intervention aimed at reaching college women: a randomized controlled trial. Prev Sci. 2012 Apr;13(2):173–82. doi: 10.1007/s11121-011-0254-1 21993613

[60] Chen VHH, Ibasco GC, Leow VJX, Lew JYY. The effect of VR avatar embodiment on improving attitudes and closeness toward immigrants. Front Psychol. 2021 Oct;12:705574. doi: 10.3389/fpsyg.2021.705574 34721153 PMC8554103

[61] Wiederhold BK. Embodiment empowers empathy in virtual reality. Cyberpsychology Behav Soc Netw. 2020;23(11):725–6. doi: 10.1089/cyber.2020.29199.editorial 33103922

[62] Rueda J, Lara F. Virtual reality and empathy enhancement: ethical aspects. Front Robot AI. 2020;7:506984. doi: 10.3389/frobt.2020.506984 33501297 PMC7805945

