# Supplementary material for: Teaching gene-environment interaction concepts with narrative vignettes: Effects on knowledge, stigma, and behavior motivation
Source: PLoS One. 2024 May 9;19(5):e0300452. doi: 10.1371/journal.pone.0300452 (PMC11081345; doi:10.1371/journal.pone.0300452)
Supplement: S1 Appendix — (DOCX) [file pone.0300452.s001.docx]

**S1 Appendix. Video Links**

**GxE Education Conditions**

GxE education: <https://vimeo.com/699038848>

Environmental framing – Bitter taste perception scenario vignette: <https://vimeo.com/699043855>

Genetic framing – Bitter taste perception scenario vignette: <https://vimeo.com/699044262>

Environmental framing – Reward based eating drive scenario vignette: <https://vimeo.com/699039141>

Genetic framing – Reward based eating drive scenario vignette: <https://vimeo.com/699039040>

**Control Condition**

Spicy food education: <https://vimeo.com/699038699>

Spicy food vignette: <https://vimeo.com/699038925>
