# Supplementary material for: Teaching gene-environment interaction concepts with narrative vignettes: Effects on knowledge, stigma, and behavior motivation
Source: PLoS One. 2024 May 9;19(5):e0300452. doi: 10.1371/journal.pone.0300452 (PMC11081345; doi:10.1371/journal.pone.0300452)
Supplement: S2 Appendix — (DOCX) [file pone.0300452.s002.docx]

**S2 Appendix. Knowledge Application Measure and Coding Document**

**Measure**

*Please read the following information about Ava and Brittany.*

*Ava has genes that make her feel full more slowly.*
*Brittany has genes that make her feel full more quickly.*

Please assume that everything else about Ava and Brittany is the same, except for their genetics. They had the same upbringing, the same taste preferences and the same metabolism.

Please read the following scenarios and estimate how many calories Ava and Brittany would eat.

**Scenario One:** Ava and Brittany are eating dinner at their own homes.

Estimate how many calories each of them consumes.

Ava: [choose on a slider scale from 0-2000]

Brittany: [choose on a slider scale from 0-2000]

Please explain why:______________

**Scenario Two:** Ava brought a prepackaged sandwich from the local deli and ate at her desk at work on Monday, and went out for lunch at a buffet on Tuesday. Imagine Ava ate 500 calories at lunch on Monday.

Estimate how many calories Ava consumed at the buffet.

Ava: [choose on a slider scale from 0-2000]

Please explain why:______________

**Scenario Three:** Brittany also brought a prepackaged sandwich from the local deli and ate at her desk at work on Monday, and went out for lunch at a buffet on Tuesday. Imagine Brittany ate 500 calories at lunch on Monday.

Estimate how many calories Brittany consumed at the buffet.

Brittany: [choose on a slider scale from 0-2000]

Please explain why:______________

**Open-ended Question Coding Document**

| **Scenario** | **Coding Explanation and Examples** |
| --- | --- |
| **Scenario 1 (Main Effect Gene)** | 1 = Explains main effect of genetic difference between Ava and Brittany (e.g., Ava will eat for longer/ Brittany feels fuller quicker.) |
|  | 0 = Absent |
| **Scenario 2 (Main Effect Environment)** | 1 = Explains main effect of environment (e.g., eat more at a buffet); MUST mention something about a buffet that logically leads to more eating (no specific mechanism required, no specific outcome required). |
|  | 0 = Absent |
| **Scenario 3 (G*E Interaction)** | 1 = Explains G*E interaction (e.g., the buffet will not influence Brittany's eating behavior as much as Ava's, or will influence it more; Environment will affect each person differently.) |
|  | 0 = Absent |
