# Supplementary material for: Teaching gene-environment interaction concepts with narrative vignettes: Effects on knowledge, stigma, and behavior motivation
Source: PLoS One. 2024 May 9;19(5):e0300452. doi: 10.1371/journal.pone.0300452 (PMC11081345; doi:10.1371/journal.pone.0300452)
Supplement: S1 File — (DOCX) [file pone.0300452.s003.docx]

**S3-S8 Tables. Omnibus Test, ANCOVAs, and Mediation Analysis Results**

**S3 Table**

*Means and Standard Deviations for Outcomes and Omnibus Test by Condition*

|  | **BTP Scenario** | | **RBED Scenario** | | **Control** | **Omnibus** | |
| --- | --- | --- | --- | --- | --- | --- | --- |
|  | **Environ**  **(*N*^1^ = 140)** | **Gene**  **(*N*^2^ = 133)** | **Environ**  **(*N* = 136)** | **Gene**  **(*N*^3^ = 136)** | **Spicy**  **(*N*^4^ = 140)** | ***F*** | ***p*** |
| **Knowledge and Belief Outcomes** |  |  |  |  |  |  |  |
| Knowledge Recall (range 0-11) | 9.21(1.60)^ab^ | 9.43(1.59)^bc^ | 9.71(1.44)^ac^ | 9.94(1.25)^c^ | 7.16(2.42)^d^ | 58.44 | **<.001** |
| Knowledge Application (range 0-6) | 4.89(1.22) | 4.80(1.24) | 4.96(1.07) | 4.86(1.14) | 4.91(1.23) | 0.31 | .87 |
| Causal Beliefs (range 1-5) |  |  |  |  |  |  |  |
| Genetic | 3.55(0.79)^a^ | 3.41(0.79)^ab^ | 3.26(0.85)^b^ | 3.52(0.77)^ab^ | 2.89(0.83)^c^ | 15.41 | **<.001** |
| Environment | 3.93(0.76) | 3.79(0.72) | 3.85(0.76) | 3.76(0.78) | 3.84(0.85) | 0.93 | .45 |
| Willpower | 3.22(0.87) | 3.35(0.91) | 3.40(0.96) | 3.26(0.97) | 3.51(0.90) | 2.20 | .07 |
| **Empathy Outcomes** |  |  |  |  |  |  |  |
| Empathetic Concern (range 1-7) | 4.28(1.57)^abc^ | 4.06(1.44)^a^ | 4.54(1.56)^ab^ | 4.70(1.37)^b^ | 3.89(1.54)^c^ | 6.75 | **<.001** |
| Perspective Taking (range 1-7) | 5.02(1.13)^ab^ | 4.95(1.22)^a^ | 5.23(1.10)^ab^ | 5.37(1.05)^b^ | 5.12(1.03)^ab^ | 3.08 | **.02** |
| **Weight Bias Outcomes** |  |  |  |  |  |  |  |
| Weight Stereotyping (range 1-5) | - | - | - | - | - |  |  |
| Negative | 2.08(0.86) | 2.22(0.83) | 2.18(0.85) | 2.14(0.76) | 2.17(0.85) | 0.51 | .73 |
| Positive | 3.06(0.81) | 3.04(0.67) | 3.06(0.76) | 3.16(0.69) | 3.01(0.74) | 0.85 | .50 |
| Weight Stigma (range 1-5) | - | - | - | - | - |  |  |
| Dislike | 1.84(0.86) | 1.87(0.86) | 1.79(0.80) | 1.71(0.75) | 1.83(0.84) | 0.81 | .52 |
| Fear | 3.21(1.26) | 3.14(1.20) | 3.21(1.11) | 3.01(1.17) | 3.07(1.24) | 0.07 | .56 |
| Willpower | 2.86(0.88) | 2.96(0.92) | 2.84(0.91) | 2.78(0.81) | 2.98(0.89) | 1.22 | .30 |
| **Behavior Change Motivation Outcomes** |  |  |  |  |  |  |  |
| Confidence (range 1-5) | 3.60(0.89) | 3.62(0.80) | 3.72(0.83) | 3.54(0.86) | 3.73(0.78) | 1.34 | .25 |
| Self-Efficacy (range 1-5) |  |  |  |  |  |  |  |
| Healthy Eating | 4.04(0.72) | 4.01(0.70) | 4.03(0.75) | 3.99(0.75) | 4.05(0.69) | 0.17 | .96 |
| Healthy Weight | 3.60(1.08) | 3.62(0.98) | 3.66(0.99) | 3.57(1.09) | 3.66(1.05) | 0.19 | .95 |
| Intention (range 1-5) | 3.20(1.09) | 3.06(1.15) | 3.11(1.17) | 3.06(1.12) | 2.98(1.15) | 0.70 | .59 |

*Notes*. ^1^ *N* = 138, ^2^ *N* = 131, ^3^ *N* = 134, and ^4^ *N* = 138 for application knowledge application. ^a–d^ Within a row, means without a common superscript differ (*p* < 0.05) due to conditions.

**S4 Table**

*One-way ANCOVA for the Main Effect of Framing*

|  | **Main Effect Framing (Genetic vs. Environmental)** | |
| --- | --- | --- |
|  | ***F*** | ***p*** |
| **Knowledge and Belief Outcomes** |  |  |
| Knowledge Recall (range 0-11) | 3.15 | .08 |
| Knowledge Application (range 0-3) | 0.87 | .35 |
| Causal Beliefs (range 1-5) |  |  |
| Genetic | 0.58 | .45 |
| Environment | 3.11 | .08 |
| Willpower | 0.00 | .96 |
| **Empathy Outcomes** |  |  |
| Empathetic Concern (range 1-7) | 0.06 | .81 |
| Perspective Taking (range 1-7) | 0.10 | .75 |
| **Weight Bias Outcomes** |  |  |
| Weight Stereotype (range 1-5) |  |  |
| Negative | 0.48 | .49 |
| Positive | 0.38 | .54 |
| Weight Stigma (range 1-5) |  |  |
| Dislike | 0.15 | .70 |
| Fear | 1.81 | .18 |
| Willpower | 0.10 | .75 |
| **Behavior Change Motivation Outcomes** |  |  |
| Confidence (range 1-5) | 1.41 | .24 |
| Self Efficacy (range 1-5) |  |  |
| Healthy Eating | 0.26 | .61 |
| Healthy Weight | 0.11 | .74 |
| Intention (range 1-5) | 1.06 | .31 |

**S5 Table**

*Two-way ANCOVAs Testing Framing-BTP Trait and Scenario-BTP Trait Interactions*

|  | **Model 1** | | | **Model 2** | | |
| --- | --- | --- | --- | --- | --- | --- |
|  | **Framing** | **BTP Trait** | **Framing* BTP Trait** | **Scenario** | **BTP Trait** | **Scenario* BTP Trait** |
| **Knowledge and Belief Outcomes** |  |  |  |  |  |  |
| Knowledge Recall (range 0-11) | 3.15 | 1.28 | 1.01 | 3.15 | 1.28 | 0.00 |
| Knowledge Application (range 0-6) | 0.87 | 1.73 | 0.03 | 0.35 | 1.73 |  |
| Causal Beliefs (range 1-5) |  |  |  |  |  |  |
| Genetic | 0.58 | 0.01 | 0.03 | 1.69 | 0.01 | 0.07 |
| Environment | 3.10 | 0.34 | 0.06 | 0.65 | 0.34 | 0.01 |
| Willpower | 0.00 | 0.81 | 0.58 | 0.38 | 0.81 | 0.16 |
| **Empathy Outcomes** |  |  |  |  |  |  |
| Empathetic Concern (range 1-7) | 0.06 | 0.19 | 0.06 | 12.15*** | 0.19 | 1.04 |
| Perspective Taking (range 1-7) | 0.10 | 0.66 | 0.90 | 10.59** | 0.66 | 2.62 |
| **Weight Bias Outcomes** |  |  |  |  |  |  |
| Weight Stereotype (range 1-5) |  |  |  |  |  |  |
| Negative | 0.49 | 9.37** | 0.90 | 0.03 | 9.35** | 0.18 |
| Positive | 0.38 | 0.02 | 0.69 | 1.09 | 0.02 | 0.01 |
| Weight Stigma (range 1-5) |  |  |  |  |  |  |
| Dislike | 0.15 | 12.26*** | 2.32 | 2.47 | 12.21*** | 0.03 |
| Fear | 1.80 | 0.01 | 0.95 | 0.40 | 0.01 | 0.15 |
| Willpower | 0.11 | 4.91* | 0.01 | 1.79 | 4.91* | 0.28 |
| **Behavior Change Motivation Outcomes** |  |  |  |  |  |  |
| Confidence (range 1-5) | 1.41 | 0.03 | 0.12 | 0.07 | 0.03 | 1.17 |
| Self Efficacy (range 1-5) |  |  |  |  |  |  |
| Healthy Eating | 0.27 | 17.58*** | 0.01 | 0.12 | 17.58*** | 0.02 |
| Healthy Weight | 0.11 | 0.75 | 0.00 | 0.00 | 0.75 | 0.34 |
| Intention (range 1-5) | 1.05 | 0.06 | 0.04 | 0.21 | 0.06 | 0.38 |

*Note*. **p* < .05, ***p* < .01, ****p* < .001. The table shows *F* values. Model 1 controlled for scenario. Model 2 controlled for framing.

**S6 Table**

*Causal Beliefs Mediating the Effect of Education Conditions (Both Genetic and Environmental Framing Conditions) Compared with Control Condition*

| **Outcome** | **Causal Beliefs** | **Condition -> Causal Belief** | **Causal Belief -> Outcome** | **Indirect effect [95% CI]** | **Direct effect** |
| --- | --- | --- | --- | --- | --- |
| Empathetic Concern | Genetic | **0.55***** | **0.42***** | **0.23 [0.14, 0.35]** | 0.23 |
|  | Environmental | -0.01 | 0.13 | -0.00 [-0.03, 0.02] |  |
|  | Willpower | **-0.20*** | **-0.23***** | **0.04 [0.02, 0.10]** |  |
| Perspective Taking | Genetic | **0.55***** | **0.24***** | **0.13 [0.06, 0.21]​** | -0.15 |
|  | Environmental | -0.01 | 0.09 | -0.00 [-0.02, 0.02] |  |
|  | Willpower | **-0.20*** | **-0.18***** | **0.04 [0.02, 0.08]​** |  |
| Negative Weight Stereotype | Genetic | **0.55***** | **-0.10**** | **-0.05 [-0.10, -0.01]​** | 0.13 |
|  | Environmental | -0.01 | -0.02 | 0.00 [-0.02, 0.01] |  |
|  | Willpower | **-0.20*** | **0.45***** | **-0.09 [-0.17, -0.02]** |  |
| Positive Weight Stereotype | Genetic | **0.55***** | **0.20***** | **0.11 [0.06, 0.16]​** | -0.03 |
|  | Environmental | -0.01 | **0.11**** | -0.00 [-0.03, 0.02] |  |
|  | Willpower | **-0.20*** | 0.02 | -0.01 [-0.02, 0.01] |  |
| Dislike (Weight Stigma) | Genetic | **0.55***** | **-0.21***** | **-0.12 [-0.18, -0.06]​** | 0.13 |
|  | Environmental | -0.01 | -0.08 | 0.00 [-0.02, 0.02] |  |
|  | Willpower | **-0.20*** | **0.19***** | **-0.04 [-0.08, -0.01]​** |  |
| Fear (Weight Stigma) | Genetic | **0.55***** | -0.07 | -0.04 [-0.11, 0.04] | 0.16 |
|  | Environmental | -0.01 | 0.06 | -0.00 [-0.02, 0.02] |  |
|  | Willpower | **-0.20*** | **0.23***** | **-0.05 [-0.10, -0.01]​** |  |
| Willpower (Weight Stigma) | Genetic | **0.55***** | **-0.32***** | **-0.18 [-0.25, -0.12]​** | **0.15*** |
|  | Environmental | -0.01 | -0.07 | 0.00 [-0.01, 0.02] |  |
|  | Willpower | **-0.20*** | **0.45***** | **-0.09 [-0.17, -0.02]​** |  |
| Confidence | Genetic | **0.55***** | **-0.09*** | **-0.05 [-0.10, -0.01]​** | 0.02 |
|  | Environmental | -0.01 | **0.11*** | -0.00 [-0.02, 0.02] |  |
|  | Willpower | **-0.20*** | **0.36***** | **-0.07 [-0.14, -0.01]​** |  |
| Healthy Eating Self Efficacy | Genetic | **0.55***** | -0.02 | -0.01 [-0.05, 0.03] | 0.00 |
|  | Environmental | -0.01 | 0.07 | -0.00 [-0.01, 0.01] |  |
|  | Willpower | **-0.20*** | **0.13***** | **-0.03 [-0.05, -0.01]​** |  |
| Healthy Weight Self Efficacy | Genetic | **0.55***** | -0.03 | -0.03 [-0.08, 0.04] | 0.04 |
|  | Environmental | -0.01 | -0.02 | 0.00 [-0.011, 0.01] |  |
|  | Willpower | **-0.20*** | **0.27***** | **-0.05 [-0.12, -0.01]​** |  |
| Intention | Genetic | **0.55***** | **0.30***** | **0.16 [0.09, 0.25]​** | 0.00 |
|  | Environmental | -0.01 | -0.00 | 0.00 [-0.01, 0.01]​ |  |
|  | Willpower | **-0.20*** | **0.18***** | **-0.04 [-0.08, -0.01]​** |  |

*Notes*. **p* < .05, ***p* < .01, ****p* < .001. The cells show unstandardized coefficients and 95% confidence intervals produced with 5,000 bootstrapping iterations.

**S7 Table**

*Causal Beliefs Mediating the Effect of Genetic Framing Condition Compared with Control Condition*

| **Outcome** | **Causal Beliefs** | **Condition -> Causal Belief** | **Causal Belief -> Outcome** | **Indirect effect [95% CI]** | **Direct effect** |
| --- | --- | --- | --- | --- | --- |
| Empathetic Concern | Genetic | **0.58***** | **0.32***** | **0.19 [0.07, 0.31]** | 0.27 |
|  | Environmental | -0.07 | 0.12 | -0.01 [-0.04, 0.02] |  |
|  | Willpower | **-0.21*** | **-0.23**** | **0.05 [0.00, 0.11]​** |  |
| Perspective Taking | Genetic | **0.58***** | **0.24***** | **0.14 [0.05, 0.23]** | -0.13 |
|  | Environmental | -0.07 | 0.13 | -0.01 [-0.04, 0.02] |  |
|  | Willpower | **-0.21*** | **-0.19**** | **0.04 [0.00, 0.10]** |  |
| Negative Weight Stereotype | Genetic | **0.58***** | -0.05 | -0.03 [-0.09, 0.03] | 0.13 |
|  | Environmental | -0.07 | -0.06 | 0.00 [-0.01, 0.02] |  |
|  | Willpower | **-0.21*** | **0.46***** | **-0.09 [-0.18, -0.01]** |  |
| Positive Weight Stereotype | Genetic | **0.58***** | **0.16***** | **0.09 [0.03, 0.16]** | 0.02 |
|  | Environmental | -0.07 | **0.10*** | -0.01 [-0.03, 0.01] |  |
|  | Willpower | **-0.21*** | 0.04 | -0.01 [-0.03, 0.010] |  |
| Dislike (Weight Stigma) | Genetic | **0.58***** | **-0.16**** | **-0.09 [-0.17, -0.03]** | 0.09 |
|  | Environmental | -0.07 | -0.08 | 0.01 [-0.01, 0.03] |  |
|  | Willpower | **-0.21*** | **0.20***** | **-0.04 [-0.09, -0.00]​** |  |
| Fear (Weight Stigma) | Genetic | **0.58***** | -0.06 | -0.03 [-0.13, 0.06] | 0.09 |
|  | Environmental | -0.07 | 0.09 | -0.01 [-0.04, 0.01] |  |
|  | Willpower | **-0.21*** | **0.23***** | **0.05 [-0.11, -0.00]** |  |
| Willpower (Weight Stigma) | Genetic | **0.58***** | **-0.32***** | **-0.18 [-0.27, -0.11]** | 0.16 |
|  | Environmental | -0.07 | -0.09 | 0.01 [-0.01, 0.03] |  |
|  | Willpower | **-0.21*** | **0.45***** | **-0.09 [-0.18, -0.01]​** |  |
| Confidence | Genetic | **0.58***** | -0.10 | -0.06 [-0.12, 0.00] | -0.02 |
|  | Environmental | -0.07 | 0.09 | -0.01 [-0.03, 0.01] |  |
|  | Willpower | **-0.21*** | **0.33***** | **-0.07 [-0.14, -0.01]​** |  |
| Healthy Eating Self Efficacy | Genetic | **0.58***** | -0.07 | -0.04 [-0.10, 0.02] | 0.02 |
|  | Environmental | -0.07 | **0.10*** | -0.01 [-0.03, 0.01] |  |
|  | Willpower | **-0.21*** | **0.11*** | **-0.02 [-0.06, -0.00]​** |  |
| Healthy Weight Self Efficacy | Genetic | **0.58***** | -0.08 | -0.05 [-0.13, 0.03] | 0.04 |
|  | Environmental | -0.07 | 0.03 | -0.00 [-0.02, 0.01] |  |
|  | Willpower | **-0.21*** | **0.25***** | **-0.05 [-0.11, -0.00]​** |  |
| Intention | Genetic | **0.58***** | **0.22**** | **0.13 [0.04, 0.23]** | -0.02 |
|  | Environmental | -0.07 | 0.00 | -0.00 [-0.02, 0.02] |  |
|  | Willpower | **-0.21*** | **0.15*** | -0.03 [-0.08, 0.00] |  |

*Notes*. **p* < .05, ***p* < .01, ****p* < .001. The cells show unstandardized coefficients and 95% confidence intervals produced with 5,000 bootstrapping iterations.

**S8 Table**

*Causal Beliefs Mediating the Effect of Environmental Framing Condition Compared with Control Condition*

| **Outcome** | **Causal Beliefs** | **Condition -> Causal Belief** | **Causal Belief -> Outcome** | **Indirect effect [95% CI]** | **Direct effect** |
| --- | --- | --- | --- | --- | --- |
| Empathetic Concern | Genetic | **0.52***** | **0.40***** | **0.21 [0.10, 0.34]** | 0.26 |
|  | Environmental | 0.05 | 0.02 | 0.01 [-0.03, 0.06] |  |
|  | Willpower | **-0.20*** | **-0.21*** | **0.04 [0.00, 0.11]​** |  |
| Perspective Taking | Genetic | **0.52***** | **0.19**** | **0.10 [0.03, 0.18] ​** | -0.14 |
|  | Environmental | 0.05 | 0.10 | 0.01 [-0.01, 0.03] |  |
|  | Willpower | **-0.20*** | **-0.15**** | 0.03 [-0.00, 0.08] |  |
| Negative Weight Stereotype | Genetic | **0.52***** | **-0.13**** | **-0.07 [-0.13, -0.02]** | 0.12 |
|  | Environmental | 0.05 | 0.00 | 0.00 [-0.01, 0.01] |  |
|  | Willpower | **-0.20*** | **0.45***** | **-0.09 [-0.18, -0.01]​** |  |
| Positive Weight Stereotype | Genetic | **0.52***** | **0.19***** | **0.10 [0.04, 0.17] ​** | -0.05 |
|  | Environmental | 0.05 | **0.14**** | 0.01 [-0.02, 0.04] |  |
|  | Willpower | **-0.20*** | 0.02 | -0.00 [-0.02, 0.02] |  |
| Dislike (Weight Stigma) | Genetic | **0.52***** | **-0.27***** | **-0.14 [-0.22, -0.07] ​** | 0.16 |
|  | Environmental | 0.05 | -0.07 | -0.00 [-0.02, 0.01] |  |
|  | Willpower | **-0.20*** | **0.15***** | **-0.03 [-0.07, -0.00]​** |  |
| Fear (Weight Stigma) | Genetic | **0.52***** | -0.11 | -0.06 [-0.15, 0.03] | 0.23 |
|  | Environmental | 0.05 | 0.12 | 0.01 [-0.02, 0.04] |  |
|  | Willpower | **-0.20*** | **0.19**** | **-0.04 [-0.09, -0.00]​** |  |
| Willpower (Weight Stigma) | Genetic | **0.52***** | **-0.33***** | **-0.17 [-0.25, -0.10] ​** | 0.13 |
|  | Environmental | 0.05 | -0.04 | -0.00 [-0.02, 0.01] |  |
|  | Willpower | **-0.20*** | **0.44***** | **-0.09 [-0.18, -0.01]​** |  |
| Confidence | Genetic | **0.52***** | -0.05 | -0.03 [-0.08, 0.02] | 0.04 |
|  | Environmental | 0.05 | 0.06 | 0.00 [-0.01, 0.02] |  |
|  | Willpower | **-0.20*** | **0.39***** | **-0.08 [-0.16, -0.01]​** |  |
| Healthy Eating Self Efficacy | Genetic | **0.52***** | 0.02 | 0.01 [-0.04, 0.06] | -0.01 |
|  | Environmental | 0.05 | 0.02 | 0.00 [-0.01, 0.02] |  |
|  | Willpower | **-0.20*** | **0.14***** | **-0.03 [-0.06, -0.00]​** |  |
| Healthy Weight Self Efficacy | Genetic | **0.52***** | 0.01 | 0.01 [-0.06, 0.08] | 0.03 |
|  | Environmental | 0.05 | **-0.16*** | -0.01 [-0.04, 0.02] |  |
|  | Willpower | **-0.20*** | **0.23***** | **-0.06 [-0.12, -0.01]** |  |
| Intention | Genetic | **0.52***** | **0.32***** | **0.17 [0.08, 0.26]​** | 0.04 |
|  | Environmental | 0.05 | 0.02 | 0.00 [-0.02, 0.02] |  |
|  | Willpower | **-0.20*** | **0.18**** | **-0.04 [-0.08, -0.00]** |  |

*Notes*. **p* < .05, ***p* < .01, ****p* < .001. The cells show unstandardized coefficients and 95% confidence intervals produced with 5,000 bootstrapping iterations.
