# Supplementary material for: Teaching gene-environment interaction concepts with narrative vignettes: Effects on knowledge, stigma, and behavior motivation
Source: PLoS One. 2024 May 9;19(5):e0300452. doi: 10.1371/journal.pone.0300452 (PMC11081345; doi:10.1371/journal.pone.0300452)

**S9-S13 Figs. Means of Outcomes Comparing Education and Control Conditions**

**S9 Figure**

*Means of Knowledge Outcomes Comparing Education and Control Conditions*


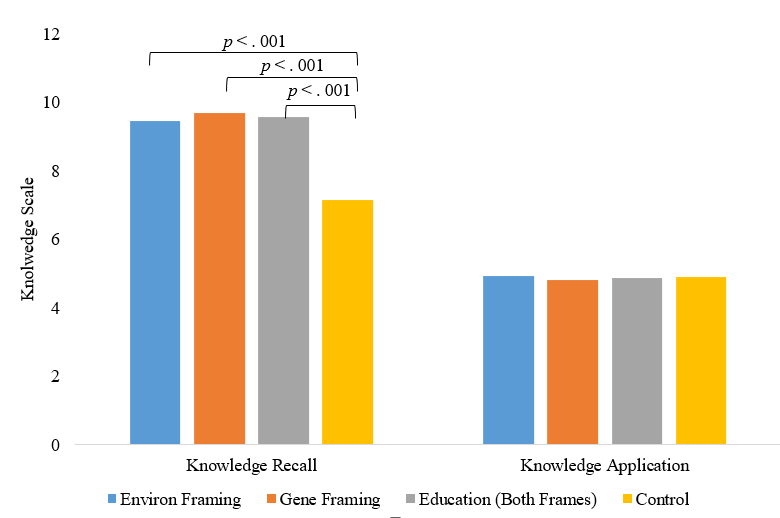


**S10 Figure**

*Means of Causal Belief Outcomes Comparing Education and Control Conditions*


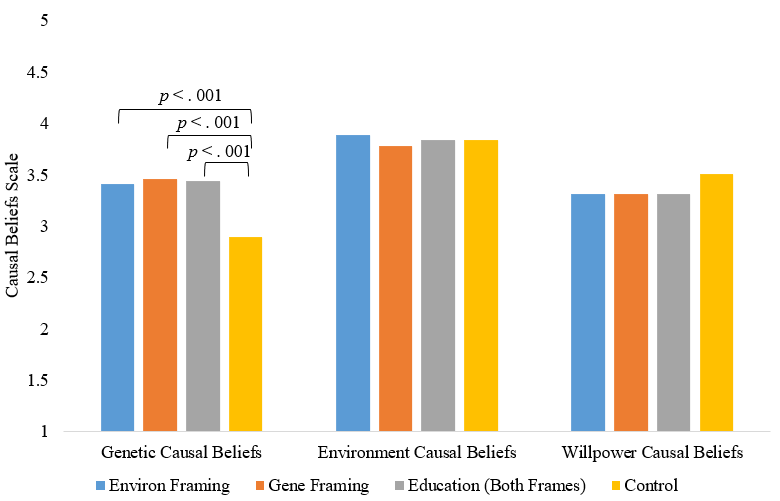


**S11 Figure**

*Means of Empathy Outcomes Comparing Education and Control Conditions*


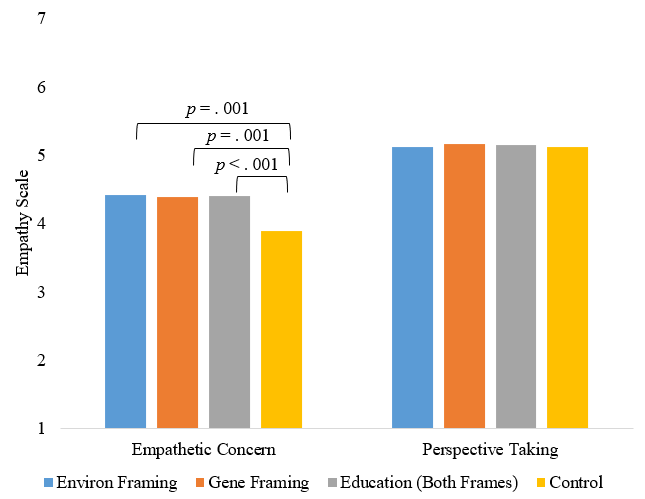


**S12 Figure**

*Means of Weight Bias Outcomes Comparing Education and Control Conditions*


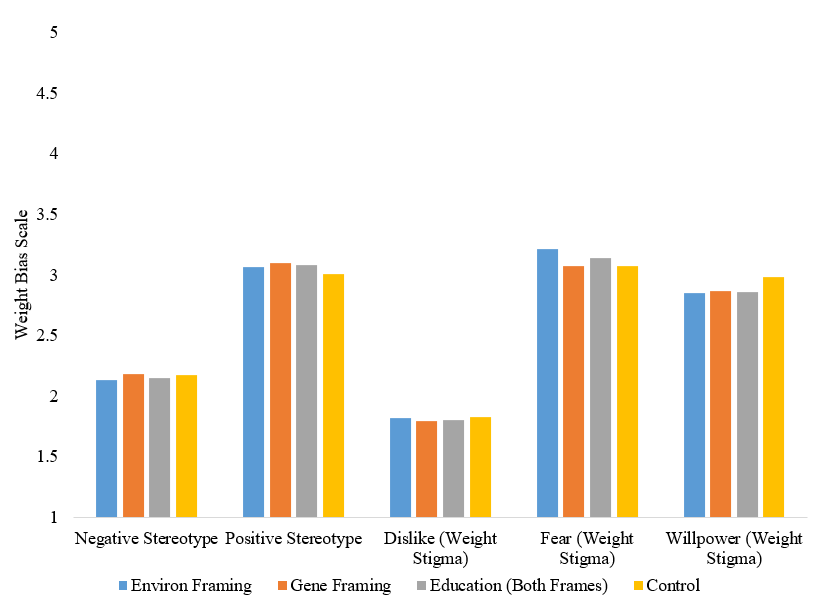


**S13 Figure**

*Means of Behavior Change Motivation Outcomes Comparing Education and Control Conditions*


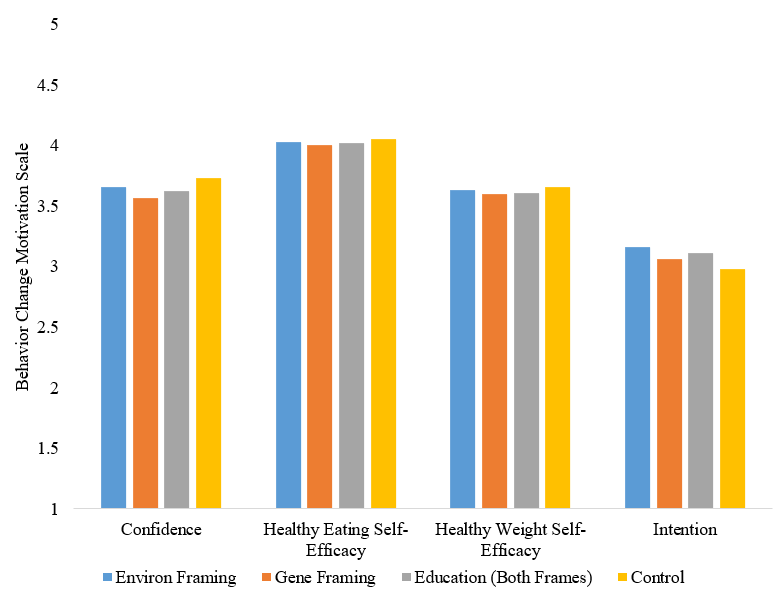

Supplement: S2 File — (DOCX) [file pone.0300452.s004.docx]
